# Benefits of insect colours: a review from social insect studies

**DOI:** 10.1007/s00442-020-04738-1

**Published:** 2020-09-02

**Authors:** Oluwatobi Badejo, Oksana Skaldina, Aleksei Gilev, Jouni Sorvari

**Affiliations:** 1 Department of Environmental and Biological Sciences, University of Eastern Finland, Yliopistonranta 1, P.O. Box 1627, 70211 Kuopio, Finland; 2 Institute of Plant and Animal Ecology (IPAE), Ural Centre of the Russian Academy of Sciences, 8 Marta Street, 202, 620144 Yekaterinburg, Russia; 3 Institute of Plant and Animal Ecology, Ural Branch, Russian Academy of Sciences, Ural Federal University, Mira Street, 19, 620002 Ekaterinburg, Russia; 4 Department of Biology, University of Turku, 20014 Turku, Finland

**Keywords:** Aposematism, Camouflage, Colouration, Thermal melanism, Hymenoptera

## Abstract

Insect colours assist in body protection, signalling, and physiological adaptations. Colours also convey multiple channels of information. These channels are valuable for species identification, distinguishing individual quality, and revealing ecological or evolutionary aspects of animals’ life. During recent years, the emerging interest in colour research has been raised in social hymenopterans such as ants, wasps, and bees. These insects provide important ecosystem services and many of those are model research organisms. Here we review benefits that various colour types give to social insects, summarize practical applications, and highlight further directions. Ants might use colours principally for camouflage, however the evolutionary function of colour in ants needs more attention; in case of melanin colouration there is evidence for its interrelation with thermoregulation and pathogen resistance. Colours in wasps and bees have confirmed linkages to thermoregulation, which is increasingly important in face of global climate change. Besides wasps use colours for various types of signalling. Colour variations of well chemically defended social insects are the mimetic model for unprotected organisms. Despite recent progress in molecular identification of species, colour variations are still widely in use for species identification. Therefore, further studies on variability is encouraged. Being closely interconnected with physiological and biochemical processes, insect colouration is a great source for finding new ecological indicators and biomarkers. Due to novel digital imaging techniques, software, and artificial intelligence there are emerging possibilities for new advances in this topic. Further colour research in social insects should consider specific features of sociality.

## Introduction

Animals use colours for diverse purposes such as body protection, signalling, and physiological adaptations (Cott 1940; Caro and Notes 2005; Cuthill et al. 2017). Colour patterns vary in shapes, luminance, tints, or polarisation and convey multiple channels of information, essential for understanding ecological and evolutionary processes, occurring in nature (Endler and Mappes 2017). Therefore, investigations of colour patterns lead to important advances in science, technology, and design (Caro et al. 2017; Cuthill et al. 2017; Schroeder et al. 2018).

Insects are the largest class of invertebrates. They are crucial for maintaining ecosystem functions and provide important ecosystem services vital to humans (Folgarait 1998; Noriega et al. 2018). Higher insect diversity favours higher functional diversity in the ecosystems, maintaining balance and sustainability. Many important ecosystem service providers (pollinators, predators, scavengers, seed dispersers) belong to hymenopteran social insects such as ants, wasps, and bees. During recent years, the increasing interest in colour research has substantially improved our understanding of colour variation in animals and led to several practical applications, particularly in social insects. Here we aim to review the functions that various aspects of colouration serve in insects with a focus on social insects and we outline the applications of assessing the colour variation of this remarkable aspect of the global biodiversity.

## Insect colouration: types and functions

### Pigment and structural colouration in insects

**Fig. 1:**
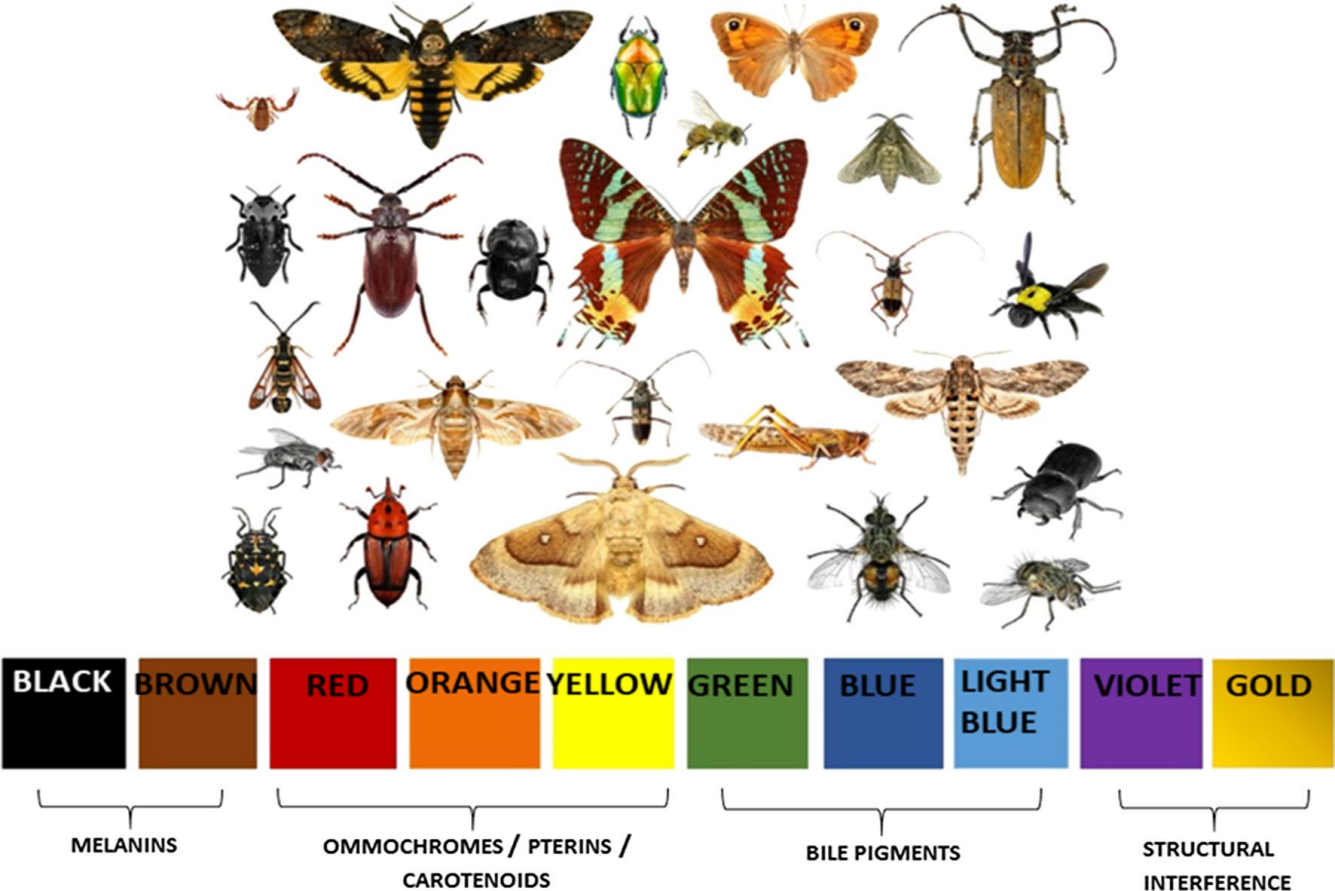
Main classes of pigment- and structure-based colours in insects. Photo © ProtasovAN

Most insects exhibit colours via absorption or reflection of sunlight using pigments, cuticular surface structures, or their combination (Chapman et al. 2013). Only some springtails, flies, and beetles possess luciferases—enzymes, which catalyze light-producing biochemical reactions, being capable of bioluminescence (Viviani 2002). Several classes of pigments are involved with insect colouration. Generally, melanins produce shades from black to reddish-brown, and pterins, ommochromes, and carotenoids contribute to red, orange, and yellow colours (Fuzeau-Braesch 1972). Furthermore, flavonoids, which are plant secondary metabolites, also create orange colours in insects (Lindstedt et al. 2010). Bile pigments or bilins result in greenish and bluish tints, violet, and golden colours often appear because of structural interference (Chapman et al. 2013) (Fig. 1). Interestingly, some insect taxa have specific pigments, like aphins, producing a variety of tints in aphids (Shamim et al. 2014) and papilochromes, resulting in yellow, orange, and red colours in butterflies (Stavenga et al. 2014). Despite all diversity, melanins and pterins are two prevalent classes of insects’ pigments (Fuzeau-Braesch 1972). Sclerotisation (hardening) and melanisation (darkening) of insect cuticle can act in conjunction, and the appearance of a colour often is the result of both processes (Andersen 2010).

Pigments are deposited in distinct places of the cuticle, which can be venation structures on the butterflies’ wings (Shvanvich 1949) or beetle elytra (Kreslavskiy 1975). Often these places are related to inner structures, such as outgrowths of the cuticle or places of the muscle attachments. For the insect species such as Colorado potato beetle (*Leptinotarsa decemlineata*), firebug (*Pyrrhocoris apterus),* and several wasp species (*Vespula* sp.) it has been shown that pigmented cuticle patterns are related to muscle bundle topography (they are places, where muscles are attached to cuticle, and the cuticle surface possess melanin spots in those places where they are attached) (Prisniy 1980; Batluzkaya 2003). Thus, insect colours depend on both pigments and structures. These structures have a morphological basis as described above or can be photonic structures, capable of interfering with visible light scale and possessing layers of the relatively high and low reflective index (Vukusic and Sambles 2003). Some specific colour tints in insects, for example, transparent white, golden-bronze, iridescent blue, or violet have only a structural basis (Fuzeau-Braesch 1972).

### Functions of colour: body protection, signalling, physiology

**Fig. 2:**
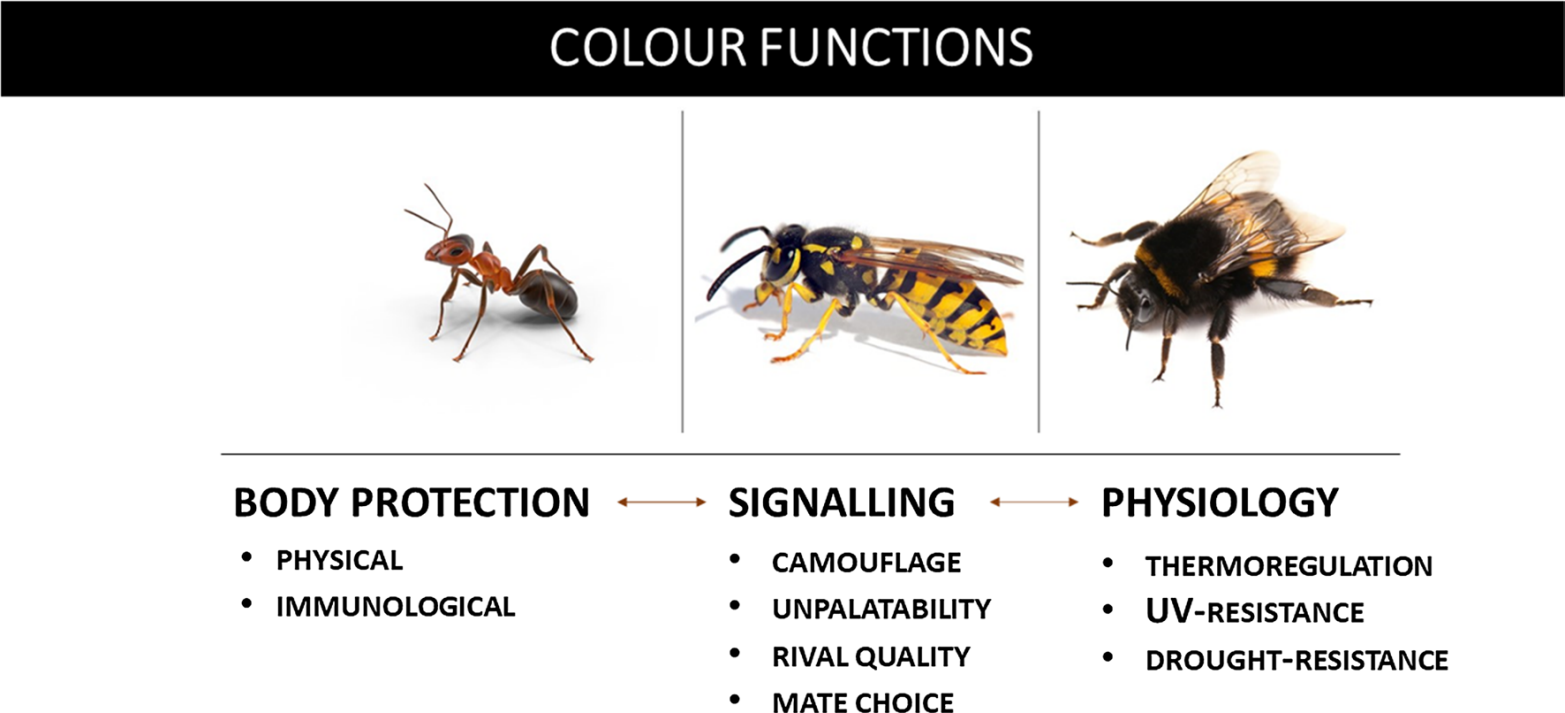
Interconnections between colour functions in social insects (ants, wasps, and bees)

**Fig. 3:**
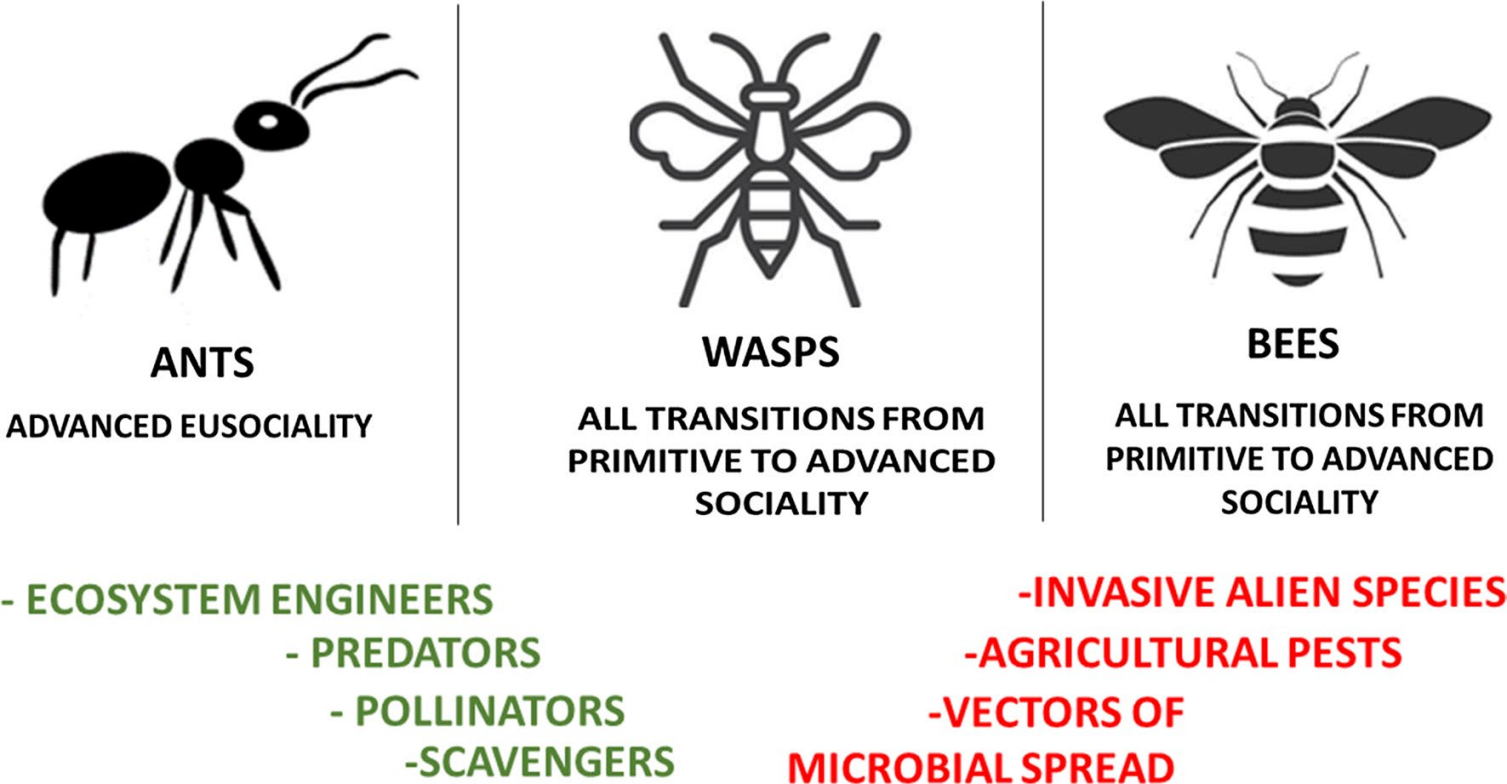
Main groups of hymenopteran social insects (ants, bees, wasps), demonstrating different levels of sociality, and diverse aspects: (1) positive (marked with green) and (2) negative (marked with red) of their ecological role

We consider the variety of colour functions in insects can be summarised as (1) body protection (physical, immunological); (2) signalling (camouflage, warning colouration, rival quality, mate choice), and (3) physiology (thermoregulation, UV-resistance, drought-resistance) (Fig. 2). In many cases, these functions are interacting, which highlights the multifunctional and integrative role of colouration. For example, camouflage and warning colouration can be referred to as both body protection and signaling (Figon and Casas 2018); or the thermoregulatory function of a colour can also possess a UV-radiation protective component. However, such a simplified classification clarifies the multifunctional and integrating role of colours in the insects’ life (Fig. 3).

Colours are related to physical and immunological body protection. Darker cuticles are generally thicker, which prevent an insect body from the penetration of pathogens and parasites (Kalmus 1941; Armitage and Siva-Jothy 2005). Melanin pigments contribute both to insects’ cuticle colouration and immunity (encapsulation response) (Gillespie et al. 1997). Therefore, melanin-based colouration can be interconnected with immunological defence. For example, in Lepidoptera moth species *Galleria mellonella*, melanic morphs were more resistant to fungal infection compared to non-melanic ones (Dubovskiy et al. 2013). Similar results were reported for the mealworm beetle *Tenebrio molitor* (Evison et al. 2017) and the mormon cricket *Anabrus simplex* (Bailey 2011). Also, an immune challenge in freshly emerged mealworm beetles reduced melanin pigment in adulthood (Kangassalo et al. 2016); which indicates a clear relationship between immunity and melanisation.

Colours help insects to hide from or scare away predators. The main mechanisms for colour-concealment are crypsis, disruptive patterning, mimesis, countershading, and counter illumination (Stevens and Merilaita 2009). From the evolutionary point of view, insect camouflage is one of the two main adaptations that reduce predation risk (Théry and Gomez 2010). The other is a warning or aposematic colouration, which signals the unpalatability of an organism to predators. Lindström et al. (2004) listed the colours that combine with black on insect species to produce aposematism, namely: red, orange, yellow, and white.

If a colour pattern reflects the physiological condition of an individual, it is considered as a true quality signal (Perez-Rodrıguez et al. 2017). Such quality signals are needed in the assessment of potential rivals or for the mate choice. Different insect species use colours for these purposes. For example, blue colours, distinguishing colouration of damselflies, require the development of bile pigments (Umbers 2013). In a damselfly *Calopteryx maculata,* blue colours were associated with fat, age, and social status of individuals (Fitzstephens and Getty 2000). Males of the blue-taled damselfly *Ischnura elegans* used colours as signals in mate choice; however, they preferred to mate with the females, possessing the colour morphs, which they have been experienced before (van Gossum et al. 2001). Many butterflies focus on wing colours and colour elements to select mate partners (Kemp and Rutowski 2011). In Colorado potato beetle, selective preference of different colour morphs for the mate choice has also been revealed (Grizenko et al. 1998). For several other beetle species, it was found that closely related species, sharing the same habitat, had noticeable colour differences associated with a quick search of a mating partner (Medvedev 1968; Kreslavskiy 1975).

Colour has beneficial physiological functions and is associated with thermoregulation, UV-light protection, and drought-resistance (True 2003; Clusella-Trullas et al. 2007; Rajpurohit et al. 2008; Roulin 2014; Pinkert and Zeuss 2018). The thermal melanism hypothesis states that individuals tend to be darker in colder environments and lighter in the warmer ones (Clusella-Trullas et al. 2007). This theory was confirmed with many examples from the insects’ world. Thus, colour variations associated with temperature were revealed in beetles (Coleoptera) (Brakefield and Willmer 1985; Korsun 2000; Schweiger and Beierkuhlein 2015); butterflies (Lepidoptera) (Zeuss et al. 2014; Stelbrink et al. 2019), dragonflies (Odonata) (Zeuss et al. 2014; Pinkert et al. 2016), parasitic wasps (Hymenoptera) (Abe et al. 2013), crickets (Orthoptera) (Fedorka et al. 2013), and in the other insects. The other environmental driving force, shaping colour variations in insects, is UV-light protection. UV-protection hypothesis states that darker individuals are better protected from insolation, and it possesses several confirming examples (True et al. 2003). The interrelation between drought-resistance and colouration in insects is also conceptualized in the melanism-desiccation hypothesis. It states that the increased melanisation favors decreasing cuticle permeability, and therefore darker individuals are better adapted to drier environments (Rajpurohit et al. 2016; Law et al. 2019). Most of the supporting evidence for this theory came from flies (Diptera) (Parkash 2010; Rajpurohit et al. 2016). To summarise, beneficial functions of colours in insects are interrelated with insect biochemical cuticle properties, colour types, life strategies, behaviour, and physiology.

### Mechanisms of colour change in insects

Both the insect colours and the colour change (an ability of an organism to modify its colour in response to specific stimuli) (Umbers et al. 2013; Figon and Casas 2018) can be used to perceive information about an organism itself or about the environment, in which that organism occurs. Some pigments like melanins, pterins, bile pigments, ommochromes are synthesized from precursors. Their effective production needs a proper physiological functioning, and in this way, pigment-based colouration is consistent with a handicap principle (Zahavi 1975) and conveys information about the quality of an organism (Stoehr 2006). The other pigments, like carotenoids, are acquired by insects from the diet. These pigments contribute more to insect physiology as antioxidants, components of short-range courtship pheromones, or as precursors for visual pigments (Heath et al. 2013). Therefore, they can signal foraging efficiency or anti-oxidative potential of an organism (Møller et al. 2000).

**Fig. 4:**
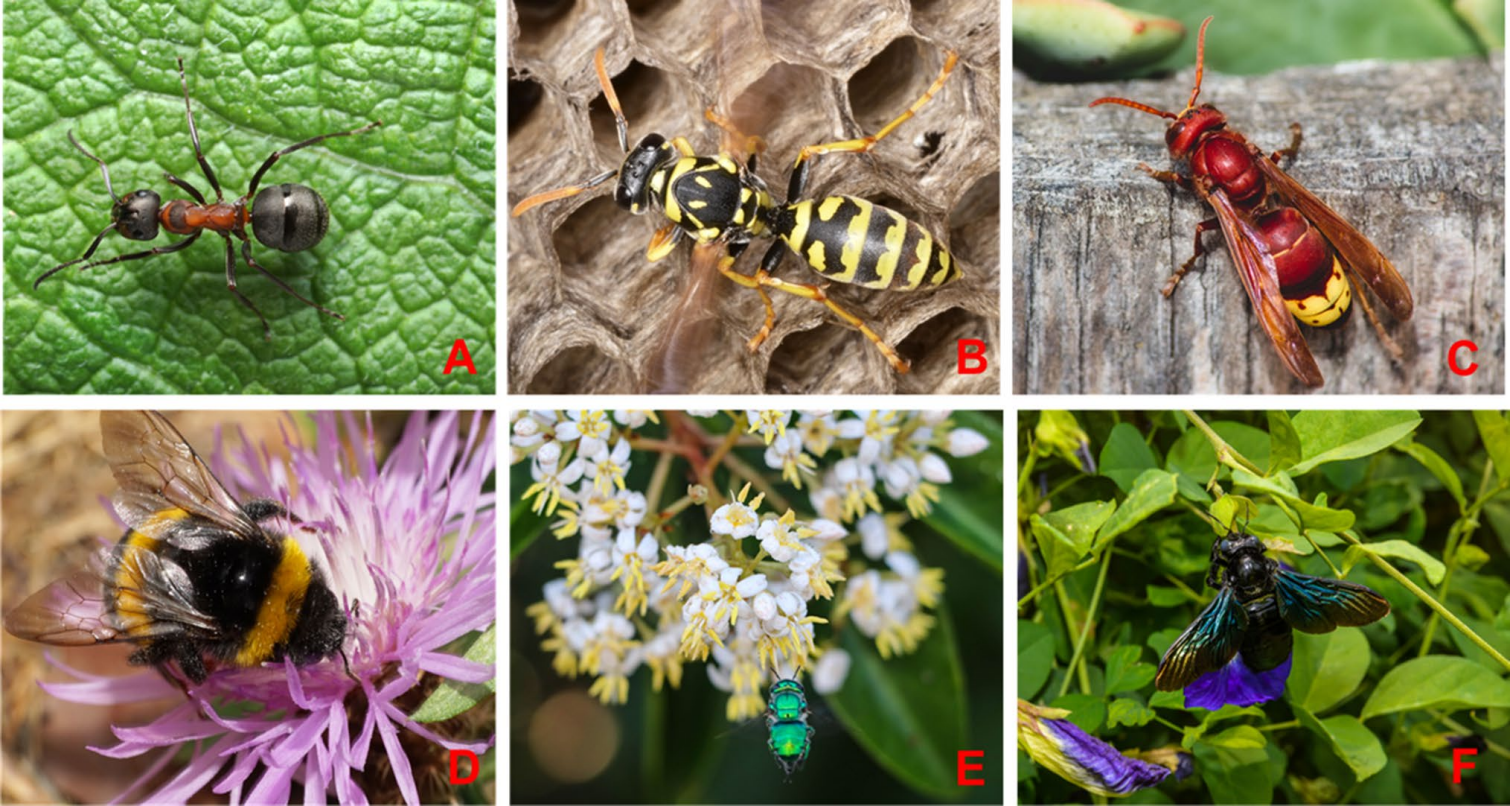
Examples of various colouration types in different species of hymenopteran insects with different levels of eusociality. **a** melanin-based pigmental black-and-red colouration of the red wood ant *Formica rufa* (highly eusocial species)*;* photo © Andrey Pavlov. **b** Melanin and pterin black- and-yellow pigment cuticular colouration of the European paper wasp Polistes dominula (primitively eusocial species); photo © Andrey Pavlov. **c** Melanin-, purine-, pteridine-based pigment colouration the Oriental hornet Vespa orientalis (highly eusocial species); photo © Tennesssee Witney **d** Melanin- and pterine-based hair colours in the White-tailed bumblebee Bombus lucorum; photo © Sergey. **e** Structural green colouration in Orchid bee Euglossa dilemma (species, exhibiting eusocial plasticity); photo © Laurel A Egan. **f** Blue pigment and structural cuticular colouration of Carpenter bee *Xylocopa* sp. (species with signs of week sociality); photo © YuRi Photolife

Mechanisms of colour change in insects are closely interconnected with beneficial functions of colours. For example, in the oriental hornet *Vespa orientalis* (Fig. 4c) yellow purine and pteridine abdominal stripes were shown to absorb solar radiation and convert it into electrochemical energy (Plotkin et al. 2009). This assists better thermoregulation in hornets and better adaptability to environmental conditions. Recently, it was revealed that the proportion of yellow abdominal pigmentation in the common wasp *Vespula vulgaris* is related to urban heat island effect (Badejo et al. 2020). These findings rise further interest to the discovering mechanisms of colour change in insects.

## Social insects: the ecosystem services providers and model organisms for research

### Social insects: definitions of a concept

Among insects, there is a specific group of organisms—“social insects”, which are of great ecological importance and represent many interesting examples related to colour functions. Belonging to two taxonomical orders Hymenoptera (ants, wasps, and bees) and Isoptera (termites), social insects comprise a group of insects with specialized castes in their colonies. These castes are based on the division of labour, sharing family members into reproductive individuals and workers, or sometimes soldiers (Brian 1983). Apart from the division of labour, the other two criteria for sociality are cooperative brood care and overlapping generations (Wilson and Hölldobler 2005). Because eusociality has evolved many times independently, hymenopterans retain representatives at different levels of sociality, ranging from subsocial, incipiently social, primitively social to advanced eusocial (Rehan and Toth 2015). Species, representing each level of sociality, differ in their major life-history traits, means of communication and the degree of reproductive isolation. Because of such striking differences and due to recent advances in molecular genetics, it has been even suggested for the primitive social insects to substitute the term “cast” into “cooperative breeders” because the females are equipotential (Sumner et al. 2018). The key traits of sociality itself, such as effective population size, level of genetic variation, and invasion success, confer several specific features on their conservation biology (Chapman and Bourke 2008). Despite that eusociality appeared in two major insect groups, it has been also reported for some thrips, beetles, and aphids (Crespi 1992; Kent and Simpson 1992; Stern et al. 1997). As here we are focused on colour benefits for social hymenopterans, we will briefly characterize each of the major groups: ants, wasps, and bees.

### Hymenopteran social insects: ants, wasps, bees

Hymenopteran social insects demonstrate different levels of sociality and provide key ecosystem services, such as pollination, biological pest control, organic matter decomposition, and seed-dispersal (Folgarait 1998; Richter 2000; Noriega et al. 2018). Simultaneously they are agricultural pests, invasive alien species, and vectors for pathogen spread (Del Toro et al. 2012; Madden et al. 2017) (Fig. 3).

There are around 16, 000 species of ants (AntWeb 2018) and all of them represent advanced eusociality (Rehan and Toth 2015). For the ecosystems, they are especially important as ecosystem engineers, which alter soil properties, make changes in nutrients and energy fluxes, and modify vegetation (Folgarait 1998; Wills and Landis 2018). They are benefitting natural ecosystems as predators and regulators of food-webs, seed spreaders, and decomposers (Folgarait 1998; Del Toro et al. 2012).

Although there are about 15, 000 aculeate (stinging) wasp species worldwide, only approximately 5% of those are eusocial and they belong to one family Vespidae (Archer 2012). Eusociality has evolved independently in the subfamilies Stenogastrinae and Polistinae/Vespinae (Hines et al. 2007). Currently, social wasps represent all possible social transitions from primitive to advanced sociality (Rehan and Toth 2015). Social wasps hunt arthropod prey and help in reducing the impact of pest species in farmlands (Gould and Jeanne 1984; Donovan 2003). They can also act as specialized pollinators (Fateryga et al. 2010; Brodmann et al. 2008) and contributors to nutrient flows (Beggs^2001^). Social wasps are stinging insects and act as pests, as they are causing damage in fruit farms or grape yards (Akre 1978), spreading polymicrobial diseases (Madden et al. 2017).

The total number of bees (Apoidea) in the world is about 20, 000 species (Michener 2000). Around 25% of this number build up a group of social bees, highly variable in the levels of sociality (Batra 1984). Among bees, tribes Apini (honeybees) and Meliponini (stingless bees) comprised of advanced eusocial species, while tribe Bombini (bumblebees) represent primitively eusocial organisms (Cardinal and Danforth 2011). Honeybees produce honey and are key pollinators of plants in both natural and agricultural landscapes. However, bumblebees and wild bees are no less important, and, in many cases, honeybees cannot substitute them for wildflowers pollination (Goulson 2003). Most of the greenhouse crops, such as tomatoes, depend on bumblebees for pollination (Kevan et al. 1991).

### Social insects as model organisms for research

A long time, social insects have been model organisms to study sociality (Sumner 2006), the evolution of complex mutualism (Gamboa et al. 2003), self-organisation, and aging (Keller and Jemielity 2006). The organised nature of colonies and the limited number of the reproductive caste provided a genetically distinct community that can be properly monitored. The ecological functions of social insects involve interaction with other components of the environment which can provide valuable information in pest management research, pollination ecology, and biomonitoring research.

## Diversity of colour functions in social insects

### The effects of social organisation on colour functions

For the social insects, specific features of sociality such as family complexity and haplodiploidy possess a decisive role for the colour function. For example, in highly eusocial ant and wasp societies, living in underground or inside constructed nests, the role of chemical cues is predominant (Akino 2008). However, recently it was found that in eusocially primitive paper wasp *Polistes dominula* there was a differential use of visual and chemical cues depending on the colony size (Cini et al. 2019). Therefore, the signalling role of colour may depend on the level of social organisation. Often there are colour differences between sexes in social insects, such as in bumblebees (Pekkarinen 1979) or *Polistes* wasps (Rusina et al. 2009). Worker ants also demonstrate conspicuous variations in colours (Skaldina and Sorvari 2017b). Interestingly, in worker red wood ants it has been shown that environmental rather than genetic driving forces (Skaldina and Sorvari 2020). That result was opposite to *Diacamma* ants (Hymenoptera, Ponerinae), in which distinct genetically based colour dimorphism was revealed between male and female genders (Miyazaki et al. 2014). We have summarized several examples of colour functions in social insects (Table 1) and further reviewed the most striking evidence of colour functions in ants, wasps, and bees.

**Table 1:** Selected examples of colour benefits for the social hymenopterans (ants, bees, wasps)

Group	Species	Benefit	Colour trait	Defined method	Reference
Ants					
Ponerinae	*Diacamma sp.*	Physical body protection associated with sexual colour dimorphism	Indices for cuticular pigmentation: black in females and yellowish-brown in males	Digital photographing photo analyses using adobe Photoshop CS5	(Miyazaki et al. 2014)
Formicinae	*Cataglyphis bombycina*	Thermoregulation	Silver tins produces by hairs	SEM and TEM imaging	(Willot et al. 2016)
Myrmicinae	*Basiceros manni*	Camouflage	Structural hairs and soil particles producing grey tints		(Hölldobler and Wilson 1986)
Bees					
Apidae/Meliponini	*Melipona costaricensis*	Thermoregulation	Light vs. dark colour	Visual assessment	(Perreboom and Biesmeijer 2003)
	*Cephalotrigona capitata*				
Apidae/Bombini	*Bombus* spp.	Thermoregulation	Light vs. dark colour	Visual assessment	
			Seven colour classes and colour-pattern elements	Coding into colour-pattern elements	(Williams 2007)
	*Bombus hortorum*	Thermoregulation	Colour forms: light, medium-light and dark	Visual assessment	(Pekkarinen 1979)
	*Bombus melanopygus*	Mimetic diversification	Pigment-based colour	Solubility test and spectrophotometry	(Hines et al. 2017)
Wasps					
Vespidae/Polistinae	*Polistes dominula*	Sexual dimorphism	Colour of the face (yellow/black)	Arttificial colour alteration	(Cappa et al. 2016)
	*Poliistes simillimus*	An assessment of rival quality in males	Proportion of black pigmentation on the head and yellow on the abdomen	Digital photographing photo analyses using Pro Plus 5.0	(de Souza et al. 2014)
	*Polistes satan*	Signal of relative fertility			
			Areas of brown and black pigmentation on the head	Digital photographing quantitative measurements	(Tannure-Nascimento et al. 2008)
	*Vespula vulgaris*	Thermoregulation			
Vespidae/Vespinae			Proportion of black pigmentation	Digital photographing quantitative measurements	(Badejo et al. 2018)
	*Vespa orientalis*	Thermoregulation	*Yellow (xanthopterin)*		
				Mass spectrometry	(Plotkin et al. 2009)

### Colours of social insects: camouflage, warning colouration, mimicry

Ants are probably the major group of social insects which utilise colours for camouflage. Different ant species are uniformly black, brown, or grey coloured, matching tree bark, soil, and rock backgrounds. However, not many studies were concentrated on colour camouflage in ants. To our knowledge, the only exception is research on the ant *Basiceros manni,* possessing specific structures: “brush” and “holding” hairs. Those structures captured and bonded soil particles and gave greyish tints to originally brown species and therefore benefitted crypsis (Hölldobler and Wilson 1986). There is also evidence for colour camouflage in bees. Thus, in dry grasslands, pale body colouration of bumblebees was suggested to have a cryptic function (Williams 2007).

In many social insects, particularly wasps and bees, but also some ant species, colouration is mainly associated with conspicuous outward appearance and poisonous mechanisms of self-defence. For example, the intensity of black-and-yellow colours in *Polistes* wasps was connected to their toxicity level (Vidal-Cordero et al. 2012). Numerous other chemically non-defended species, like flies, beetles, and spiders, started to mimic social insects in their appearance and behaviour (Brian 1983). This type of mimicry is named Batesian; and mimetic species are highly variable in the degree of their similarity to models (Edmunds 2000). Pérez-Espona et al. (2018) studied *Ecitomorpha* and *Ecitophya* beetle species which were protected from predation through mimicry (body shape and colour) to the army ant *Eciton burchellii*. On the other hand, there are some examples when less chemically defended social insects mimic those which are stronger protected. For example, Australian carpenter ant *Camponotus bendigensis*, is mimetic to the Jack Jumper bull ant *Myrmecia fulvipes*, possessing powerful sting and capable for jumping on short distances (Merrill and Elgar 2000). Another *Camponotus* sp*.* imitates conspicuous black-and-yellow colouration of poisonous ant *Crematogaster inflata* (Ito et al. 2004). Besides, social insects exhibit the second type of mimicry. The observed multiple colours of bumblebees have developed into Müllerian mimicry when several chemically well-defended species possess similar phenotypic traits (Stiles 1980; Williams 2007; Hines and Williams 2012).

### Colours of social insects: individual quality and mate choice

Among social insects, signalling function of colour was the most fully studied for the wasps. It was shown that paper wasps *Polistes* sp. used colours for the assessment of the rival’s quality, for the mate choice, or individual recognition (Tibbetts and Dale 2004; Tibbetts 2010; Tibbetts et al. 2014). Prior to engaging in competitive fights and with the aim to protect their nests *P. gallicus,* foundresses assessed social status of their rivals using colour signals (Petrocelli et al. 2015). Highly variable size and shape of clypeal marking in *P. dominulus* were shown to predict dominant status of females (Tibbetts et al. 2011; Tibbetts and Injaian 2014). In *P. satan* Tannure-Nascimento et al. (2008) showed that female brown facial colour patterns were important for the establishment of linear hierarchy during a nest founding stage. Those facial colour traits were linked to the relative fertility of the foundresses and their capability for successful nest maintenance. Interestingly, in *Polistes* wasps, it was found that females of different colour morphotypes had substantial differences in the mode of nest foundation (Rusina et al. 2007). This might be also related to the role of colour in social interactions among wasps (Tibbetts and Dale 2004; Tibbetts 2006).

Also, social insects use colour for mate choice. It has been shown that in *Polistes simillimus*, the larger was the proportion of black pigmentation on the head of a male, the higher was the chance of getting a mating partner (de Souza et al. 2014). Male colouration was similarly identified as a mediator of sexual selection in *P. dominulus* (Izzo and Tibbetts 2012). In this species, yellow abdominal spots in males were proposed to be sexual signals, as females preferred males with smaller and eclipse-shaped yellow spots. In the other species from that genus (*P. nimphus* and *P. gallicus*) colour traits were linked with reproductive strategies (Rusina et al. 2019). Interestingly, while assessing the fighting ability, *P. dominula* males also used colour patterns (de Souza et al. 2016a). Cappa et al. (2016) showed that individuals of the wasp species *P. dominula* use facial colour markings for sexual interaction and gender identification.

### Colours of social insects: thermoregulation, UV-resistance, drought-resistance

Colouration of social insects is toughly interconnected with their physiology and in some species has proven connections to thermoregulation, humidity-, and UV-light adaptations. Recent findings indicate that the colour lightness (melanisation) of ant assemblages is mainly driven by thermoregulation (Bishop et al. 2016) and UV-resistance (Law et al. 2019). For the Australian ant *Iridomyrmex purpureus*, a distinct difference in colour forms concerning humidity was established. The “typical” morph preferred wet conditions, while the unusual “blue” morph was more adapted to the dry habitats (Greenslade 1976). In North American ant species *Formica neorufibarbis gelida,* inhabiting mountain tundra, dark small workers foraged in the morning, when there was a lack of sunlight and heating. While light preferred foraging in the afternoon, when there was an excess of insolation (Bernstein 1976). The thermoregulatory and UV-protective functions of colour was also revealed for the wasps. For example, in *Polistes* wasps, darker individuals were found to be associated with colder climatic zones (de Souza et al. 2016b). The occurrence of darker individuals in higher latitude was described for the common wasp *Vespula vulgaris* (Badejo et al. 2018) and social paper wasp *Agelaia pallipes* (de Souza et al. 2020). In stingless bees, dark coloured individuals warmed up faster than light-coloured ones (Pereboom and Biesmeijer 2003). The authors also linked the distribution of stingless bees with their colouration, as in high latitudes species were completely black, which demonstrated the involvement of colour into thermoregulation. A similar trend was earlier described in the bumble bees (Pekkarinen 1979). Less information is available for the relationships between colour and immunity, but de Souza et al. (2011) and showed that the melanisation of the *Carpenter* ant *Camponotus* fellah increases in response to antibiotic treatment.

## The assisting role of social insects’ colouration

### The role of colour for species identification

Various groups of social insects, including bees (Hines and Williams 2012), wasps (Dvořák and Roberts 2006; Dvořák et al. 2012), and ants (Na and Lee 2001) are identified using colour variations on the different body parts. For example, Guerrero and Fernandez (2008) used colour traits for the description of a new species—the ant *Forelius damiani* in Colombia. Similarly, Sosa-Calvo et al. (2010) used colouration of ant species in Guyana for species identification. Colour of the mandible (whitish), antennae and legs (yellowish), head and gaster (dark-brown or black) as well as waist segments (light brown) were main distinguishing characteristics of *Strumigenys royi* (Formicidae, Myrmicinae, Attini). On one hand, Dlussky et al. (1998) showed that head colouration can be used for a reliable delimitation of fire ant species. On the other hand, investigation on the colour traits of the Central Asian *Lepisiota* (= *Acantholepis*) species allowed combining several previously described species into a polymorphic one—*A. semenovi* (Dlussky 1981). However, in some cases, species identification using colour patterns was not reliable for distinguishing cryptic species (Carolan et al. 2012; Vesterlund et al. 2014).

### The use of colour traits in ecological surveys and biomonitoring

Various colour traits in different organisms were proposed as effective and non-invasive indicators of environmental stress (Lifshitz and St Clair 2016). Earlier in the current review, we summarised that several colour traits in social insects are sensitive to environmental conditions and reflect the individual performance. Such biological responses are useful for the development of ecological indicators and novel biomarkers in social insects (Skaldina and Sorvari 2017c). For example, we have found that red wood ants *Formica lugubris* have less melanised heads in environments polluted with heavy metals (Skaldina et al. 2018). Similarly, common wasps *Vespula vulgaris* possessed decreased areas of melanised facial colour markings in industrial areas, and that decrease was associated with certain metal elements (Skaldina et al. 2020). Colouration of the ant species *Formica aquilonia* was sensitive to forest clear-cutting (Skaldina and Sorvari 2017a). Therefore, with proper further validation colour traits can assist biomonitoring and make some environmental surveys cheaper and more convenient.

### The use of colour for research assisting

Discovering insect colouration has several research-assisting aspects. Those are revealing the range of intra- and within-species variability, biochemical properties of pigments and biophysical properties of nanostructures, discovering insects’ age or a specific physiological condition, related to colouration.

One of the first studies about wasps’ colouration showed diverse within and inter-species variability in North American species of social wasps (Enteman 1904). Since that time, detailed studies on colouration variability in several genera of wasp species such as *Vespa* (Perrard et al. 2014), *Vespula* (Clapperton et al. 1989; Badejo et al.^2018^), *Dolichovespula* (Eck 1981, 1983) and *Polistes* (Tibbetts et al. 2011) have been performed. Workers of *Formica aquilonia* showed colour variation across Fennoscandia and some parts of Russia due to the species’ postglacial recolonisation pathways (Gilev et al.^2015^). Based on the signs of colour, the intraspecific phenotypic differentiation of ants *Formica* s. str. had been revealed at different scales (Gilev et al. 2006; Antonov and Gilev 2016). Such variations can be used in diverse ecological and evolutional research.

The other research direction is a biochemistry of insect pigments and biophysics of cuticle structure. There are many cases of pigment-based integument or structural colouration in these group of social insects, as ants and wasps’ colours are mainly distinguished by pigments (Fig. 4a, b). Pigments, present in hairs, distinguish colouration of numerous bees and bumblebees, like for instance *Bombus lucorum* (Fig. 4d). However, there is also structural colouration in social insects. For example, metallic-green colouration in primitively eusocial Orchid bee *Euglossa dilemma* (Fig. 4e) or dark-blue pigment colouration of carpenter bees *Xylocopa* sp. (mainly solitary insects with signs of primitive eusociality; Fig. 4f). Concerning unusual colouration, Nemésio (2005) for the first time found evidence for fluorescence in Neotropical orchid bee *Eulaema niveofasciata*, however, the question about its evolutionary meaning remains unclear.

In a stingless bee *Melipona quadrifasciata* tergite, pigmentation was studied as a marker of hypopharyngeal glands to facilitate certain aged worker capturing (Fagundes et al. 2006). In red wood ants, smaller workers are generally younger and darker than bigger (and older) ones (Gilev 2003; Skaldina and Sorvari 2017b). Therefore, using colour traits as markers of the insect age or status has promising future research avenues.

## Future of the social insect colour studies

### Current progress in methods

Methods to assess insect coloration range from image analysis of standardised pictures of specimens and drawings as well as spectral imaging to utilization of advanced software, which allows processing different channels of information simultaneously (Lehnert et al. 2011). Images can be obtained using a combination of camera and several microscopy methods including stereomicroscopes (Guerrero and Fernández 2008; Sosa-Calvo et al. 2010), scanning electron microscopes (SEM), spectrophotometers (Stelzer et al. 2010) and Raman spectrometry (RS) (Carlo et al. 2017). The useful software for image processing is referred to as both well-known programs such as Photoshop, ImageJ, Pro Plus 5.0 (De Souza et al. 2014; Miyazaki et al. 2014; Skaldina et al. 2017a) and new software specially designed to study colour traits, such as WaspFacer (Skaldina et al. 2020). Furthermore, current computer-assisted image analysis allow utilising readily available images which allows conducting large-scale colour research projects (see e.g., Stelbrink et al. 2019). With the development of photographing techniques, the colour research, based on processing museum collections has received a new rise (de Souza et al. 2016b).

### Knowledge gaps and further research directions

Despite substantial progress, our review revealed several knowledge gaps in the topic. First, very little information is available about evolutionary function of colouration in ants and bees. Second, almost nothing is known about the interrelation between melanisation and pathogen resistance in social insects. Structural and combined types of colouration are much less discovered in comparison to pigment colour type. Finally, the majority of colour studies in social insects are referred to melanin-based colourations, and there is a need to study the other types.

Our renewed scientific interest in insect colouration raises new questions and opens several research topics. In the face of global environmental change (and primarily climate change), the adaptive function of colouration towards thermal conditions and UV-radiation is of special importance. New studies on the relationship between colour and temperature in both model and non-model insects and further practical monitoring actions, based on these studies, will help to preserve biodiversity, and maintain environmental sustainability. Current development of databases and artificial intelligence techniques will create further possibilities for conducting large-scale projects. On the other hand, local scale studies, measuring insect colours and investigating their changes through time will favor our understanding of the ecological process ongoing in certain populations. Insect colours should receive greater attention for the development of novel biomonitoring tools and as the research-assisting purposes. The other promising research direction of insect colouration is design of bioinspired biomaterials. The variety of possibilities to develop new functional materials using examples from the insect world is reviewed by Schroeder et al. (2018). Further progress of this research direction might lead to new advances in design and engineering. Further colour research in social insects should consider specific features of sociality.

## Conclusions

The history of colours in the animal kingdom has fascinated biologist early on. Pigment, structural, and combined colour types possess many useful functions. Those are (1) physical and immunological body protection; (2) signalling, including camouflage, unpalatability, rival quality, and mate choice; (3) physiological adaptations to temperature, UV-radiation, and desiccation. Available studies allow assuming that among ants, colours are needed principally for camouflage and thermoregulation. In wasps, colour variations have diverse signalling purposes and possess confirmed interrelations with thermoregulation. Climate-related colour variations were also found in bees. However, evolutionary function of colouration in ants and bees remains one of the knowledge gaps. The current rapid development of databases, digital software, and artificial intelligence allows creation the large-scale colour research projects of high ecological importance. Further discovering colouration variability in social insects might favour progress in species identification, biomonitoring, and other research fields such as environmental ecology, biochemistry, biophysics, and biomaterial design.
